# Lipid-lowering prescription patterns after a non-fatal acute coronary syndrome: A retrospective cohort study

**DOI:** 10.1016/j.ijcrp.2025.200385

**Published:** 2025-03-06

**Authors:** Cristina Gavina, Daniel Seabra Carvalho, Marta Afonso-Silva, Inês Costa, Ana Sofia Freitas, Mariana Canelas-Pais, Nuno Lourenço-Silva, Tiago Taveira-Gomes, Francisco Araújo

**Affiliations:** a Cardiology Department, Hospital Pedro Hispano–Unidade Local de Saúde Matosinhos, Matosinhos, Portugal; b Department of Medicine, Faculty of Medicine of University of Porto, Porto, Portugal; c UNIC, Faculty of Medicine of University of Porto, Porto, Portugal; d Real World Evidence Department, Novartis Farma–Produtos Farmacêuticos SA, Porto Salvo, Portugal; e Medical Department, Novartis Farma–Produtos Farmacêuticos S.A., Porto Salvo, Portugal; f MTG Research and Development Lab, Porto, Portugal; g Department of Community Medicine, Information and Health Decision Sciences (MEDCIDS), Faculty of Medicine, University of Porto, Porto, Portugal; h Faculty of Health Sciences, Fernando Pessoa University (FCS-UFP), Portugal; i CINTESIS@RISE Center for Health Technology and Services Research, Porto, Portugal; j SIGIL Scientific Enterprises, Dubai, United Arab Emirates; k Department of Internal Medicine, Hospital Lusíadas, Lisbon, Portugal

**Keywords:** Atherosclerosis, Hydroxymethylglutaryl-CoA reductase inhibitors, Low-density lipoprotein cholesterol, Electronic health records

## Abstract

**Background:**

After an acute atherosclerotic cardiovascular event, high-intensity lipid-lowering therapy (LLT) is needed to reduce recurrence risk. This study aimed to describe LLT prescription patterns and LDL-C levels change after non-fatal acute coronary syndrome (ACS) events and to determine if the recommended goals for LDL-C levels were achieved.

**Methods:**

Retrospective cohort study using electronic health records (EHR) of Unidade Local de Saúde de Matosinhos between 2015 and 2023. Participants were adults aged 40–80 years, with a non-fatal ACS hospitalization between 2016 and 2022 (index date); ≥1 general practice appointment in the three years before ACS; and one-year follow-up post-ACS. Sub-analyses focused on gender, age (<and ≥65 years), and whether patients met LDL-C control (55 mg/dL) at one-year post-ACS.

**Results:**

Of 544 patients, 270 (49.6 %) were under 65 years, and 164 (30.1 %) were females. Before the ACS, 71.1 % of men and 56.7 % of women had no previous LLT prescription and younger patients showed poorer LDL-C control (132(IQR 64)mg/dL) than older patients (102(IQR 50)mg/dL). One-year post-ACS, only 11.3 % of males and 8.5 % of females met LDL-C target. The proportion of patients without LLT decreased from 66.7 % at baseline to 13.6 % post-ACS. High-intensity LLT prescriptions increased from 2.4 % to 16.5 %, while moderate-intensity LLT remained predominant (65.8 %). Still, 89.5 % of patients had uncontrolled LDL-C levels.

**Conclusion:**

Despite initiating/intensifying LLT, one year after ACS most patients did not achieve LDL-C goals. This indicates a significant gap in guideline implementation in clinical practice.

## Highlights

• Retrospective study using EHR from a health integrated unit between 2015 and 2023.
• 544 adult patients were identified and followed-up during 1-year post-ACS.
• Patients without LLT decreased from 66.7 % at baseline to 13.6 % post-ACS.
• Few patients achieved LDL-C targets post-ACS, suggesting room to optimize LLT.

## 1 Introduction

Atherosclerotic Cardiovascular Disease (ASCVD) is a major worldwide health concern and has been the subject of numerous research efforts and public health strategies in recent decades [1]. Based on the most recent information from the World Health Organization (WHO), ischemic heart disease ranks as the top global death cause, accounting for over a third of total deaths [2]. ASCVD contributes significantly to disability-adjusted life years and premature deaths globally [3]. Recent data from Portugal showed that it is responsible for over 32,000 annual fatalities [4,5]. Thus, mitigating its modifiable risk factors becomes pivotal [6].

The 2019 guidelines by the European Society of Cardiology/European Atherosclerosis Society (ESC/EAS) for the management of dyslipidemias categorized patients into four distinct cardiovascular (CV) risk levels: low, moderate, high, and very high; and for each defined different low-density lipoprotein cholesterol (LDL-C) control levels. Numerous risk factors, including hypertension, diabetes, obesity and smoking, contribute to individual CV risk and increase the risk for recurrent CV events [7,8]. This classification is intended to assist in determining the best treatment and monitoring plan for each individual [9]. Using a population cohort from the city of Matosinhos in Northern Portugal, our group is studying Lipid mAnagemenT IN pOrtugal (LATINO Study) and has previously reported that those at higher CV risk face a greater likelihood of death and ASCVD hospital admissions than those at lower risk [10,11], supporting previous findings [[12], [13], [14]].

The management of patients after acute coronary syndrome (ACS) has evolved significantly, with major randomized controlled trials like IMPROVE-IT, FOURIER, and ODYSSEY-Outcomes offering evidence-based guidance on therapeutic approaches [[15], [16], [17], [18]]. The IMPROVE-IT trial demonstrated that adding ezetimibe to simvastatin in patient post-ACS significantly reduced CV events, emphasizing the role of intensive lipid-lowering therapy (LLT) [16]. Similarly, the FOURIER trial highlighted the efficacy of the PCSK9 inhibitor evolocumab in reducing the risk of CV events in patients with established CV disease, including those with a history of ACS, further underscoring the benefit of aggressive LDL-C reduction [18]. The ODYSSEY Outcomes trial provided evidence that alirocumab, another PCSK9 inhibitor, significantly reduced the risk of recurrent CV events in post-ACS patients with elevated LDL-C levels despite statin therapy, offering an alternative for patients who require further LDL-C lowering [17]. Collectively, these trials support the implementation of intensive lipid-lowering strategies, including the inhibition of PCSK9 in addition to statins, to reduce recurrent CV events in the post-ACS context. Given this, experts now recommend the early initiation of combination therapies due to evidence of the beneficial effects of aggressive and rapid LDL-C reduction following an event, moving from a wait-and-see approach to adopting combination therapy as the foundational standard of care for patients at very high and extremely high risk [19].

Thus, this study aimed to describe LLT prescription patterns and LDL-C levels change after non-fatal ACS events and to determine if the ESC/EAS recommended goals for LDL-C levels were achieved.

## 2 Methods

### 2.1 Study design

A retrospective and incident study was conducted using electronic health records (EHRs) of Unidade Local de Saúde de Matosinhos (ULSM), Portugal between 01-01–2015 and 31-12-2023. Selected participants in the research had to fulfill these criteria: (i) non-fatal ACS hospitalization between 01-01–2016 and 31-12-2022 (index date); (ii) at least one general practice appointment in the three years prior to the index date; (iii) age range between 40 and 80 years at index date; and (iv) a documented record in the year after the index date (365 + 30 days). These inclusion criteria maximize the overlap of the study population with the resident population, which accounts for approximately 90 % of the resident population of Matosinhos, according to the latest 2021 Portuguese Census [20].

### 2.2 Data access and patient characterization

Access to data for analysis was authorized following the approval from the Ethical Committee and Data Protection Officer at Unidade Local de Saúde de Matosinhos (ULSM), under the approval code N.°21/CE/JAS on February 12, 2021. Anonymized demographic and clinical data were gathered from EHRs in compliance with the Health Insurance Portability and Accountability Act (HIPAA) Safe Harbor provisions [21] for patient profiling. Identification of all pertinent health conditions was achieved through the use of respective codes from the International Classification of Primary Care, 2nd edition (ICPC-2), the International Classification of Disease-9th Revision (ICD-9), and the International Classification of Disease-10th Revision (ICD-10). Specific vocabularies standardized to Systemized Nomenclature of Medicine – Clinical Terms (SNOMED CT) were employed for coding laboratory and clinical measurements and drugs were mapped in line with the Anatomical Therapeutic Chemical (ATC) Classification System. Source data was adjusted to fit the Observational Medical Outcomes Partnership - Common Data Model 5.4.1 standard. Source data was converted into a final dataset for analysis, allowing for statistical evaluations detailed in the findings.

The categorization of statin intensity, influenced by medication type and dosage, adhered to the standards of the American College of Cardiology and the American Heart Association [21]. Four LLT classifications were established: low to high LLT based on statin intensity and the inclusion of ezetimibe (as detailed in Supplementary material) and no LLT based on the absence of prescription of these therapies. Both combined and individual pill formulations of LLTs were taken into account. If patients modified their statin type or dosage but stayed within a similar intensity bracket, their LLT category remained unchanged.

### 2.3 Cohort definition

**Fig. 1:**
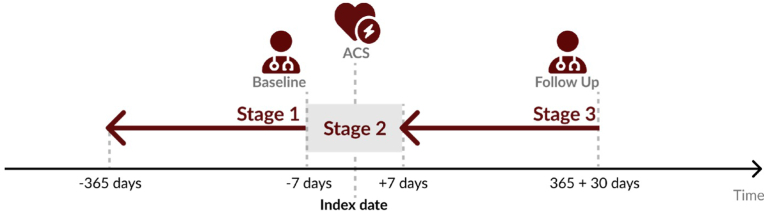
Study design. Index date: non-fatal ACS event between 01-01–2016 and 31-12-2022. Stage 1- maximum period of 365 days before the index date (data closer to index date was used); Stage 2- period of 7 days before and 7 days after the index date (data closer to index date was used); Stage 3- one year (365 + 30 days) after index date (last data available during this follow-up period).

The ACS cohort included only patients with non-fatal ACS events (unstable angina or myocardial infarction)(index date). Defined time points for this incident study were set at the period of maximum 365 days before the index date (stage 1), period of 7 days before and 7 days after the index date (stage 2), and one year (365 + 30 days) after index date (stage 3) (Fig. 1). Stage 1 aimed to record patients' sociodemographic and clinical characteristics before the ACS event. Stage 2 aimed to evaluate LDL-C levels and LLT prescriptions near the event. It was defined as a period between 7 days before and 7 days after the non-fatal ACS event to ensure we had an LDL-C level immediately in the peri-ACS period. In this stage, we also analyzed the changes in LLT prescriptions that occurred near the ACS event. In stage 3, we evaluate the last record of LDL-C level and LLT prescriptions during the one year after the ACS event.

### 2.4 Outcomes

The outcomes evaluated at each stage were LLT prescription (frequency for each LLT intensity level) and LDL-C level (median value of LDL-C levels). It was estimated the proportion of patients below the threshold level defined for each stage: <100 mg/dL (2.6 mmol/L) at stage 1; <70 mg/dL (1.82 mmol/L) at stage 2 and < 55 mg/dL (1.4 mmol/L) at stage 3. The threshold for stage 3 was established on the premise that patients post-ACS, having confirmed coronary heart disease, are classified as being at very-high-risk for subsequent CV events. Thus, the goal for LDL-C levels in these patients is set at <55 mg/dL(<1.4 mmol/L), according to the 2019 ESC/EAS guidelines [9,15]. Thresholds for stage 1 and stage 2 were defined based on clinical local experience, with stage 2 characterized by a linear decrease from the period before the ACS event (an approximate 50 % reduction).

### 2.5 Statistical analysis

Basic descriptive statistics were used to present the global characteristics of the study population. Sub-analyses were performed by age group (<65 years and ≥65 years), by gender, and by LDL-C control rates at stage 3. The continuous variables were reported with median and interquartile range (IQR). Categorical variables were presented as absolute and relative frequencies. We used Apache Spark Framework version 3.5.0 to engineer the final dataset, R version 4.3.2 to perform the statistical analysis, and Vega-lite to generate figures [22,23].

## 3 Results

### 3.1 Patient characteristics

The demographic characteristics of the total cohort and of each defined subgroup (gender, age group, LDL-C control at stage 3) are shown in Table 1. This study included 544 patients post-ACS, of whom 30.1 % (n = 164) were females and 49.6 % (n = 270) younger than 65 years. The median age of all patients was 65 (IQR 16) years. The median age was fairly consistent between genders, with males having a slightly lower median age of 63 years. When grouped by age, the <65 years group had a median age of 56 (IQR 10) years, while the ≥65 years group had a median age of 72 (IQR 8) years. Regarding gender distribution, in the <65 years group, females constituted 22.6 % (n = 61) of the population, whereas in the ≥65 years group, women comprised 37.6 % (n = 103).

**Table 1:** Patients’ demographic and clinical characteristics (n = 544).

Patient Characteristics	Total	Gender		Age group		LDL-C Control at stage 3	
		Male	Female	<65 years	≥65 years	Controlled	Uncontrolled
Number of patients, n	544	380	164	270	274	57	487
Female, n (%)	164 (30.1)	–	–	61 (22.6)	103 (37.6)	14 (24.6)	150 (30.8)
Age, years, P50 (IQR)	65 (16)	63 (16)	68 (16)	56 (10)	72 (8)	68 (14)	64 (17)
**LDL-C (mg/dL) at time points**							
LDL-C at stage 1, P50 (IQR)	116 (62)^a^	116 (66)	113 (52)	132 (64)	102 (50)	90 (45)	120 (62)
LDL-C at stage 2, P50 (IQR)	94 (48)^b^	93 (46)	97 (48)	97 (46)	94 (48)	52 (21)	98 (46)
LDL-C at stage 3, P50 (IQR)	88 (41)^c^	88 (41)	90 (40)	89 (42)	85 (40)	46 (8)	92 (38)
**LDL-C control at time points**							
LDL-C Controlled at stage 1, n (%)	191 (35.1)^a^	134 (35.3)	57 (34.8)	65 (24.1)	126 (46.0)	34 (59.7)	157 (32.3)
LDL-C Controlled at stage 2, n (%)	108 (19.9)^b^	78 (20.5)	30 (18.3)	43 (15.9)	69 (25.2)	43 (75.4)	67 (15.0)
LDL-C Controlled at stage 3, n (%)	57 (10.5)^c^	43 (11.3)	14 (8.5)	20 (7.4)	37 (13.5)	57 (100.0)	(0.0)
**LLT Medications at stage 1**							
No LLT, n (%)	363 (66.7)	270 (71.1)	93 (56.7)	204 (75.5)	159 (58.0)	37 (64.9)	325 (66.9)
Low-intensity LLT, n (%)	23 (4.2)	15 (3.9)	8 (4.9)	7 (2.6)	16 (5.8)	4 (7.0)	19 (3.9)
Moderate-intensity LLT, n (%)	145 (26.7)	85 (22.4)	60 (36.6)	55 (20.4)	90 (32.9)	16 (28.1)	129 (26.5)
High-intensity LLT, n (%)	13 (2.4)	10 (2.6)	3 (1.8)	4 (1.5)	9 (3.3)	0 (0.0)	13 (2.7)
**LLT Medications at stage 2**							
No LLT, n (%)	70 (12.9)	59 (15.5)	27 (16.4)	28 (10.4)	42 (15.3)	6 (10.5)	67 (13.7)
Low-intensity LLT, n (%)	32 (5.9)	20 (5.3)	8 (4.9)	9 (3.3)	23 (8.4)	4 (7.0)	28 (5.7)
Moderate-intensity LLT, n (%)	374 (68.8)	261 (68.7)	112 (68.3)	193 (71.5)	181 (66.1)	38 (66.7)	333 (68.5)
High-intensity LLT, n (%)	68 (12.5)	40 (10.5)	17 (10.4)	40 (14.8)	28 (10.2)	9 (15.8)	59 (12.1)
**LLT Medications at stage 3**							
No LLT, n (%)	74 (13.6)	50 (13.1)	24 (14.6)	35 (12.9)	39 (14.2)	3 (5.3)	70 (14.4)
Low-intensity LLT, n (%)	22 (4.0)	14 (3.7)	8 (4.9)	8 (3.0)	14 (5.1)	3 (5.3)	19 (3.9)
Moderate-intensity LLT, n (%)	358 (65.8)	248 (65.3)	110 (67.1)	183 (67.8)	175 (63.9)	42 (73.6)	317 (65.0)
High-intensity LLT, n (%)	90 (16.5)	68 (17.9)	22 (13.4)	44 (16.3)	46 (16.8)	9 (15.8)	81 (16.7)

Relative frequencies represent valid percentages. IQR – Interquartile range; LDL-C – low-density lipoprotein cholesterol; LLT – Lipid-lowering therapy; P50 – Median. Stage 1- period of 365 days before the index date; Stage 2- period of 7 days before and 7 days after the index date; Stage 3- one year (365 + 30 days) after index date. Index date: non-fatal ACS event between 01-01–2016 and 31-12-2022.a- 537 valid cases; b- 540 valid cases; c – 543 valid cases.

### 3.2 Variation of LDL-C levels and LLT prescription

**Fig. 2:**
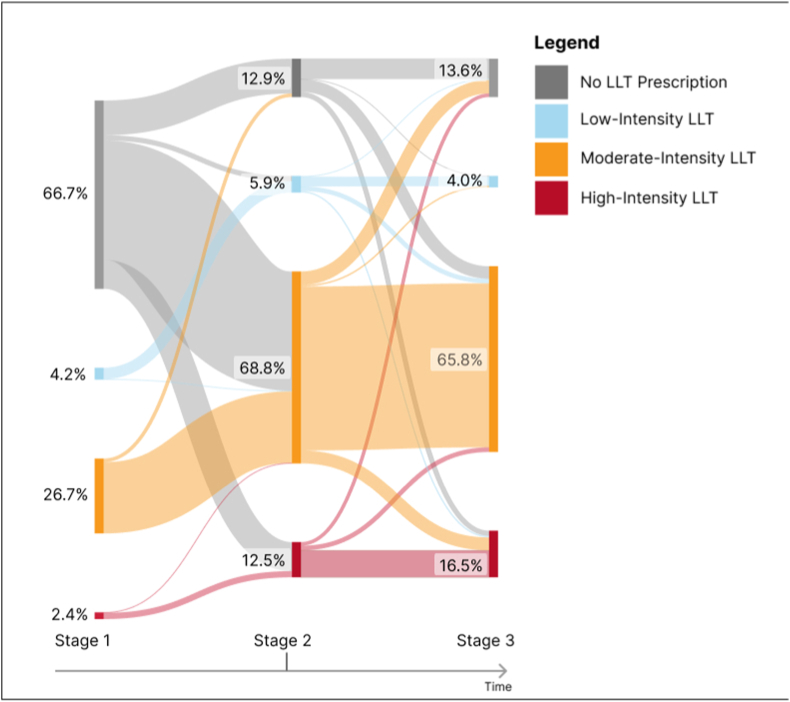
Proportion of patients in each Lipid-Lowering Therapy (LLT) intensity level through the three stages. Stage 1- period of 365 days before the index date; Stage 2- period of 7 days before and 7 days after the index date; Stage 3- one year (365 + 30 days) after index date. Index date: non-fatal ACS event between 01-01–2016 and 31-12-2022.

Fig. 2 depicts the variation of the LLT regimens for the whole population included in the study, showing a shift towards moderate-intensity and high-intensity LLT in both stages 2 and 3. Overall, at stage 2, 68.8 % (n = 374) of patients had a prescription of moderate intensity LLT and 12.5 % (n = 68) of high-intensity LLT. By stage 3, the usage patterns of LLT medications remained consistent with stage 2, with a slight increase in high-intensity LLT (n = 90; 16.5 %). Description of variation of LDL-C levels and LLT prescription for each subgroup are shown in Table 1 and described below.

### 3.3 Gender

Median LDL-C level peaked at stage 1, with only a slight difference between men and women (116 (IQR 66) mg/dL versus 113 (IQR 52) mg/dL, respectively. One-year post-ACS (stage 3), both genders showed a decline to their lowest median LDL-C levels of 88 (IQR 41) mg/dL and 90 (IQR 40) mg/dL for males and females, respectively. Analysis of LDL-C control over the three stages demonstrated a pronounced decline in the proportion of patients maintaining controlled LDL-C levels. At stage 1, the proportion of patients with controlled LDL-C levels was relatively balanced between genders, with 35.3 % of males (n = 134) and 34.8 % of females (n = 57). By stage 2, there was a notable decrease in LDL-C control among the patients as the control rate dropped to 20.5 % for males (n = 78) and was even more pronounced among females (18.3 %; n = 30). One-year post-ACS (stage 3), only 11.3 % of male patients (n = 43) and 8.5 % of female patients (n = 14) had their LDL-C levels within the recommended range.

**Fig. 3:**
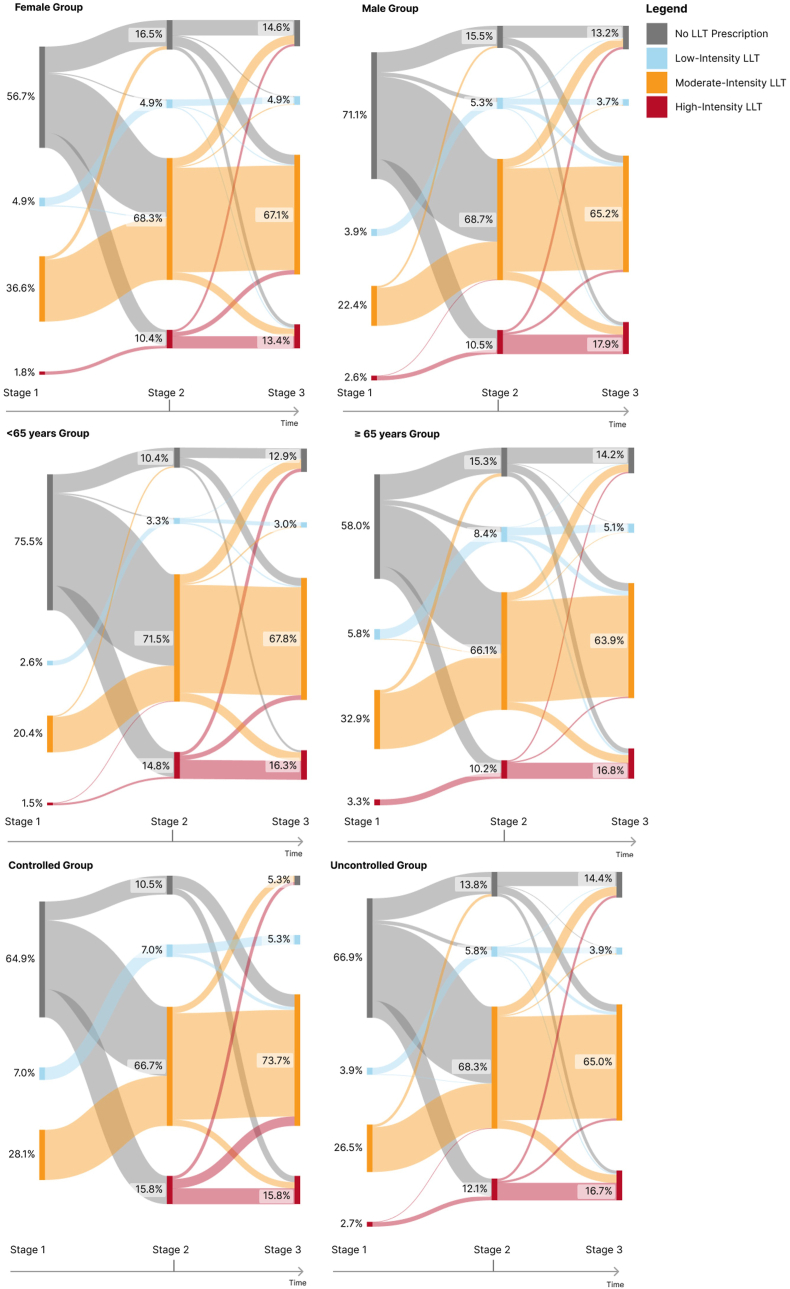
Proportion of patients in each Lipid-Lowering Therapy (LLT) intensity level through the three stages, categorized by gender (male, female), age (<65 years and ≥ 65 years) and LDL-C control at stage 3. Stage 1- period of 365 days before the index date; Stage 2- period of 7 days before and 7 days after the index date; Stage 3- one year (365 + 30 days) after index date. Index date: non-fatal ACS event between 01-01–2016 and 31-12-2022.

When examining LLT prescriptions during these time frames, the data revealed some differences between men and women, as seen in Fig. 3. Specifically, 71.1 % of men and 56.7 % of women had not been prescribed any form of LLT before the ACS event. The sub-analysis highlighted a shift in LLT medication use post-ACS, namely an increase in the use of moderate (males from 22.4 % to 68.7 %, females from 36.6 % to 68.3 %) and high-intensity LLT (males from 2.6 % to 10.5 %, females from 1.8 % to 10.4 %).

### 3.4 Age groups

For the <65 years group, the median LDL-C started at 132 (IQR 64) mg/dL and decreased to 89 (IQR 42) mg/dL during the follow-up period, whereas, for the older group, the starting median LDL-C was 102 (IQR 50) mg/dL, reducing to 85 (IQR 40) mg/dL over time. The <65 years group showed higher LDL-C levels at baseline (stage 1) and, consequently, a lower proportion of controlled patients than the older patients (24.1 % versus 46.0 %, respectively). At stage 3, older patients exhibited better control than their younger counterparts (13.5 % versus 7.4 %, respectively).

There were notable differences among the two groups regarding the prescription of LLT at stage 1. A total of 75.5 % (n = 204) of the patients in the <65 years group and 58.0 % (n = 159) of patients in the ≥65 years group did not take any LLT. The moderate intensity LLT was the most common LLT regimen, with 20.4 % (n = 55) and 32.9 % (n = 90) in the <65 years and ≥65 years groups, respectively. High-intensity LLT was the least common prescription regimen in both groups in stage 1. At stage 2, 71.5 % and 66.1 % of patients <65 and ≥65 years, respectively, had a prescription of moderate intensity LLT; and over a tenth (10.4 % and 15.3 % for patients <65 and ≥65 years, respectively) of the population did not have any prescription. One year after the event, the disparities in prescription patterns between the age groups became negligible. The analysis of Fig. 3 can help to better understand the variations of LLT over the 3 stages under evaluation.

### 3.5 LDL-C control at stage 3

Only 10.5 % (n = 57) patients had their LDL-C levels at stage 3 within the recommended range, which composed the controlled group. The median LDL-C levels for all patients started high (90 (IQR 45) mg/dL for the controlled group and 120 (IQR 62) mg/dL for the uncontrolled group), but showed a decline throughout the subsequent time stages. When comparing these two cohorts in terms of the proportion of patients achieving LDL-C targets, there are notable differences. Regarding the uncontrolled group, the highest recorded proportion of participants with controlled LDL-C levels is recorded before the ACS event (32.3 %, n = 157).

At stage 1, the proportion of LLT prescriptions between the controlled and uncontrolled groups did not show many differences. In the controlled group, moderate intensity LLT was the most prescribed type of LLT (28.1 %, n = 16), and 64.9 % (n = 37) of patients did not have any kind of LLT prescription. Only slight differences were observed between groups one-year post-ACS, with the moderate intensity LLT being the most frequent regimen (65.0 % of the uncontrolled group and 73.6 % of the controlled group). The number of patients without an LLT prescription was low, especially in the controlled group (5.3 % versus 14.4 % in the uncontrolled). Fig. 3 provides a better understanding of the LLT prescription patterns and visualizes how they vary over the three time periods.

## 4 Discussion

This study aimed to analyze how ACS influenced the control of LDL-C levels and the prescription of LLT. Our work demonstrated that, at one-year post-ACS, LDL-C values decreased under LLT in all patient groups but most patients failed to achieve goals set by the current guidelines. The potential occurrence of an ACS is closely linked to unregulated LDL-C levels. The DYSlipidemia International Study (DYSIS), involving several countries, including Portugal, reported inadequate control of lipid levels in patients undergoing statin treatment [24,25], a finding mirrored by the DISGEN-LIPID study [26]. The DA VINCI study found that only 33 % of patients achieved the target LDL-C level as per ESC/EAS 2019 guidelines [27], and even poorer results were reported by the LATINO study, with less than 5 % achieving their risk-based goals [11]. A comprehensive study from Sweden recently evaluated the correlation between fluctuations in LDL-C and the strength of statin treatment in predicting outcomes after a heart attack. The study found that a significant early decrease in LDL-C levels and a more aggressive statin regimen following a heart attack were linked with a lower risk of CV events and death from any cause [28].

A predominant pattern emerged in the period prior to the ACS event (stage 1). In every group, a significant majority (exceeding 66.7 %) had no LLT prescription. Moderate-intensity LLT dominated the prescription charts, with proportions oscillating between 20.4 % for the young demographic and 36.6 % for females. Works by Yen-Ting Yeh et al. and Fox, M. Kathleen et al. showed similar findings, also verifying that there are more males included in the ACS study and that most patients had a moderate intensity LLT prescription [29,30]. At stage 2, LLT prescription experienced a shift, as seen in other studies [31,32]. The prescription of moderate-intensity LLT corresponded to about two-thirds of prescriptions of the study participants, and there was a decrease in the proportion of patients without any LLT prescription, settling at 10.4 % for the young cohort and 16.5 % for females. When we transitioned into the follow-up period (stage 3), the proportion of patients without an LLT prescription was similar to the stage 2, with the exception of the controlled group, marking a decline to 5.3 %, which might explain the LDL-C control rate of this group. The highest proportion of high-intensity LLT prescriptions peaked during this period; however, moderate-intensity LLT prescriptions still held their position as the predominant choice. Although there was a slight increase in the amount of high-intensity LLT prescriptions, the overall proportion of LLT prescriptions remained almost unchanged between the period of the event and the follow-up period, except for the controlled cohort.

The shift in LLT prescriptions may have favored the decrease of LDL-C levels evident in all groups, but without a pronounced effect, it did not allow the achievement of recommended LDL-C goals for patients at very-high CV risk (according to ESC/EAS 2019 guidelines). Kristensen, M. et al. also referred to the poor LDL-C goals among patients who suffered an ACS [31]. This reality can be attributed not only to the slow adoption of guidelines in clinical practice but also to factors such as patient non-adherence and therapeutic inertia [33].

This study has some limitations that should be acknowledged. We assessed LLT regimens based on prescription records, although not differentiating between primary and secondary care prescriptions. It was not possible to confirm whether prescriptions were filled, we have a limitation regarding whether patients adhere to the therapy. Studies like Fox, M. Kathleen et al. showed a significant discontinuation rate over the follow-up period [29]. Although other studies like Ersbøll et al. have observed an increase in LDL-C control and LLT intake [34], this is not the case in our study. However, this study differed from ours in relation to the LDL-C target over time (70 mg/dL) and also the duration of the follow-up period (2 years). In addition, the use of health care resources, such as number of appointments with general practitioners or with other specialists after the non-fatal ACS could have been also described. All patients analyzed were classified as having very-high CV risk due to the occurrence of a non-fatal ACS event. However, it would have been also valuable to describe the presence of other cardiovascular risk factors, including hypertension, diabetes, obesity, and smoking. Generalization of these results to other populations is limited as these data are relative to a specific population in the north of Portugal. However, the high rate of patient reconstruction of a stable regional population provides a detailed and unconstrained real-world perspective of patient populations under primary and secondary care followed in similar settings.

## 5 Conclusion

LDL-C values decreased, under LLT, in all patient groups after ACS, however most patients failed to achieve goals set by current guidelines. There is a need to optimize LLT in clinical practice to reduce ASCVD and cardiovascular-related mortality, tailoring LLT prescriptions for patients and LDL-C level goals.

## CRediT authorship contribution statement

**Cristina Gavina:** Writing – review & editing, Supervision, Methodology, Conceptualization. **Daniel Seabra Carvalho:** Writing – review & editing, Conceptualization. **Marta Afonso-Silva:** Writing – review & editing, Conceptualization. **Inês Costa:** Writing – review & editing. **Ana Sofia Freitas:** Writing – review & editing. **Mariana Canelas-Pais:** Software. **Nuno Lourenço-Silva:** Writing – review & editing, Writing – original draft, Validation, Software, Methodology, Formal analysis. **Tiago Taveira-Gomes:** Writing – review & editing, Validation, Supervision, Software, Methodology, Conceptualization. **Francisco Araújo:** Writing – review & editing, Supervision, Conceptualization.

## Ethical statement

The database use was authorized by the Ethical Committee and Data Protection Officer of ULSM (translated from *Comissão de Ética para a Saúde da Unidade Local de Saúde de Matosinhos*) under approval codes N.°21/CE/JAS.

The results presented in this article have not been published previously in whole or part.

## Data availability statement

All aggregate statistical results are incorporated into the article and its online supplementary material. Patient-level data was not used in this study and is not publicly available.

## Funding sources

This study was conducted within the scope of a collaboration protocol in clinical research between Novartis Farma - Produtos Farmacêuticos SA, and Sociedade Portuguesa de Aterosclerose.
