# Supplementary material for: Lipid-lowering prescription patterns after a non-fatal acute coronary syndrome: A retrospective cohort study
Source: Int J Cardiol Cardiovasc Risk Prev. 2025 Mar 6;25:200385. doi: 10.1016/j.ijcrp.2025.200385 (PMC11929879; doi:10.1016/j.ijcrp.2025.200385)
Supplement: Multimedia component 1 [file mmc1.docx]

Lipid-Lowering Prescription Patterns After a Non-Fatal Acute Coronary Syndrome: a retrospective cohort study

Supplementary Material

Contents

[Local Health Unit of Matosinhos constitution 2](#_Toc192175454)

[Supplementary Tables 4](#_Toc192175456)

[Table S1 4](#_Toc192175457)

[Table S2 5](#_Toc192175458)

[Table S3 6](#_Toc192175459)

[Table S4 7](#_Toc192175460)

[Table S5 8](#_Toc192175461)

[Table S5 9](#_Toc192175462)

# Local Health Unit of Matosinhos constitution

Local Health Unit of Matosinhos (ULSM) provides primary, differentiated and continuous healthcare. Its area of influence corresponds to the municipalities of Matosinhos, Vila do Conde and Póvoa de Varzim.

## ULSM Units

- **Pedro Hispano Hospital**
- **Health Care Center Leça da Palmeira**
  - Family Health Unit Leça
  - Family Health Unit Maresia
  - Family Health Unit Dunas
  - Family Health Unit Progresso
  - Primary Care Unit Santa Cruz
  - Continuity Care Unit Leça
- **Health Care Center Matosinhos**
  - Family Health Unit Horizonte
  - Family Health Unit Oceanos
  - Primary Care Unit Matosinhos
  - Continuity Care Unit Matosinhos
- **Health Care Center Senhora da Hora**
  - Family Health Unit Caravela
  - Family Health Unit Lagoa
  - Family Health Unit Custóias
  - Continuity Care Unit Senhora da Hora
- **Health Care Center S. Mamede de Infesta**
  - Family Health Unit Infesta
  - Family Health Unit Porta do Sol
  - Primary Care Unit S. Mamede
  - Continuity Care Unit S. Mamede

# Supplementary Tables

## Table S1

Definitions of risk according to 2019 ESC/EAS Guidelines

| **Variable** | **Definition** |
| --- | --- |
| Low risk | SCORE < 1% |
| Moderate risk | At least one is true:   1. T1DM before age 35 2. T2DM before age 50 for <10 years without risk factors 3. SCORE within [1,5[% |
| High risk | At least one is true:   1. TC > 310 mg/dL 2. LDL-C > 190 mg/dL 3. SBP ≥ 180mmHg or DBP ≥ 110mmHg twice ≥ 7 days 4. FH 5. DM with no target organ damage and either    1. Another CV Risk Factor    2. Duration ≥ 10 years 6. eGFR within [30, 60[ mL/min 7. SCORE within [5, 10[ % |
| Very high risk | At least one is true:   1. MI, UA, SA, PCI, CABG, STK, TIA, or PAD 2. DM with target organ damage 3. At least 3 Major CV Risk Factors 4. eGFR < 30 mL/min 5. T1DM duration > 20 years 6. SCORE ESC 19 ≥ 10% 7. FH with either ASCVD or Major CV Risk Factor |

ASCVD - atherosclerotic cardiovascular disease; CABG - coronary artery bypass graft surgery; CV - cardiovascular; DBP - diastolic blood pressure; DM - diabetes mellitus; eGFR: estimated glomerular filtration rate; EAS: European Atherosclerosis Society; ESC: European Society of Cardiology; FH: familial hypercholesterolemia; LDL-C: low-density lipoprotein cholesterol; MI: myocardial infarction; SBP: systolic blood pressure; SCORE: systematic coronary risk estimation; PAD: peripheral arterial disease; PCI: percutaneous coronary intervention; SA: stable angina; STK: stroke; T1DM, type 1 diabetes mellitus; T2DM, type 2 diabetes mellitus; TC: total cholesterol; TIA: transient ischemic attack; UA: unstable angina.

## Table S2

Risk-based LDL-C goals according to 2019 ESC/EAS Guidelines

| **Risk category** | **LDL-C goal** |
| --- | --- |
| Low | A goal of <116 mg/dL (<3.0 mmol/L) |
| Moderate | A goal of <100 mg/dL (<2.6 mmol/L) |
| High | A goal of <70 mg/dL (<1.8 mmol/L) * and a >50% reduction from baseline |
| Very high | A goal of <55 mg/dL (<1.4 mmol/L) * and a >50% reduction from baseline |

EAS - European Atherosclerosis Society; ESC - European Society of Cardiology; LDL-C - low-density lipoprotein cholesterol; * Only absolute target levels were implemented.

## Table S3

Definitions of lipid lowering treatment (LLT) categories

| **Variable** | **Definition** |
| --- | --- |
| Statin | Patients treated with any statin (with or without other LLT) |
| Low intensity statin | Patients treated with any low intensity statin (with or without other LLT) |
| Moderate intensity statin | Patients treated with any moderate intensity statin (with or without other LLT) |
| High intensity statin | Patients treated with any high intensity statin (with or without other LLT) |
| Ezetimibe + Statin | Patients treated with ezetimibe plus any statin (low/moderate/high intensity statin) |
| Ezetimibe + Low intensity statin | Patients treated with ezetimibe plus low intensity statin |
| Ezetimibe + Moderate intensity statin | Patients treated with ezetimibe plus moderate intensity statin |
| Ezetimibe + High intensity statin | Patients treated with ezetimibe plus high intensity statin |
| Ezetimibe monotherapy | Patients treated with ezetimibe (with or without other LLT) |
| PCSK9 inhibitors | Patients treated with PCSK9i (evolocumab) |
| Fibrates | Patients treated with fibrates |
| Other | Alipogene, bempedoic acid, bile acid sequestrants, dextrothyroxine, inclisiran, lopitamide, meglutol, mipomersen, pyridoxal, policosanol, probucol, nicotinic acid derivatives, omega 3, tiadenol |

LLT - Lipid lowering treatment.

## Table S4

Definitions of statin intensity categories

| **Variable** | **Definition** |
| --- | --- |
| Low intensity statin | Simvastatin 10 mg or Pravastatin 10–20 mg or Lovastatin 20 mg or Fluvastatin 20–40 mg or Pitavastatin 1 mg |
| Moderate intensity statin | Atorvastatin 10–20 mg or Rosuvastatin 5–10 mg or Simvastatin 20–40 mg or Pravastatin 40–80 mg or Lovastatin 40 mg or Fluvastatin XL 80 mg or Fluvastatin 40 mg BID or Pitavastatin 2–4 mg |
| High intensity statin | Atorvastatin 40–80 mg or Rosuvastatin 20–40 mg |

##

## Table S5

Lipid-lowering therapy (LLT) intensity categories

| **Variable** | **Definition** |
| --- | --- |
| No LLT | Neither statin nor ezetimibe |
| Low intensity LLT | Low intensity statin |
| Moderate intensity LLT | Low intensity statin + ezetimibe or Moderate intensity statin |
| High intensity LLT | Moderate intensity statin + ezetimibe  High intensity statin  High intensity statin + ezetimibe |

## Table S5

Study variables definitions

| **Variable** | **Definition** |
| --- | --- |
| Age | Patient age in years |
| Alcohol or Drug Abuse | At least one is true:   - ICD-9: 305 - ICD-10: F101, F19 - ICPC-2: P15, P16, P19 |
| Antithrombotic Agents | ATC: B01 |
| Atherosclerotic disease | Meets criteria for UA, MI, ischemic STK, or PAD |
| Atrial fibrillation | At least one in true:   - ICD-9: 4273 - ICD-10: I48 - ICPC-2: K78 |
| Beta Blockers | ATC: C07, C09BX02, C09BX04, C09BX05 |
| Calcium Channel Blockers | ATC: C08 |
| Cancer | At least one is true:   - ICD-9: 14, 15, 16, 17, 18, 19, 20, 21, 22, 23 - ICD-10: C - ICPC-2: B74, N74, D74, D75, D76, L71, T71, R84, S77, Y77, U75, U76, U77, W72, X75, X76, X77 |
| Cardiovascular disease | Meets criteria for SA, UA, MI, AF, STK, HTN, CKD, PAD, or TIA |
| Cardiovascular death | Hospital episode that resulted in death during that episode with the episode bearing at least one:   - ICD-9: 41-43 - ICD-10: I |
| Chronic Immune Inflammatory Disorder | At least one is true:   - ICD-9: 710, 714, 555, 556 - ICD-10: M3, M05, M06, M07, M08, M09, M10, M11, M12, M13, M14, K50, K51 - ICPC-2: L88, D94 |
| Chronic kidney disease | At least one measurement of eGFR ≥ 60 mL/min followed by two measurements of eGFR < 60 mL/min, the latter at least 90 days apart. CKD is staged from the latest measurement |
| Chronic Obstructive Pulmonary Disease | At least one is true:   - ICD-9: 496 - ICD-10: J44 |
| Coronary heart disease | Meets criteria for UA, MI, PCI, or CABG |
| Corticosteroids | ATC: H02 |
| CVD Risk level ESC 19 | CVD Risk level as defined in 2019 European Cardiology Society Guidelines for Dyslipidaemias (Low Risk, Intermediate Risk, High Risk, Very High Risk) |
| Definite Familial hypercholesterolemia | At least one is true:   - ICD-9: 2720 - ICD-10: E7801 |
| Death | Date of death is registered for the patient |
| Diuretics | ATC: C03 |
| Familial hypercholesterolemia | Meets criteria for Definite FH or Possible FH |
| Glucose Lowering Drugs | ATC: A10 |
| Haemorrhagic stroke | At least one is true:   - ICD-9: 43[0-2] - ICD-10: I6[012] |
| Heart failure | At least one is true:   - ICD-9: 404[019]3, 428 - ICD-10: I110, I130, I132, I50 - ICPC-2: K77 |
| Human Immunodeficiency Virus Infection | At least one is true:   - ICD-9: 042 - ICD-10: B20 - ICPC-2: B90 |
| Hypercholesterolemia | Total Cholesterol > 190 mg/dL |
| Hypertension | At least one is true:   - ICD-9: 413, 4140 - ICD-10: I201, I208, I209, I251, I255 - ICPC-2: K76 - SBP >140 mmHg or DBP >90 mmHg twice >7 days |
| Ischemic stroke | At least one is true:   - ICD-9: 43[34678] - ICD-10: I6[356] |
| LDL-C Control ESC 19 | Patients with LDL-C levels under the recommended 2019 ESC/EAS target according with CVD risk level |
| Mental Health Disorder | At least one is true:   - ICD-9: 29, 30, 31 - ICD-10: F[0-9] - ICPC-2: P70, P71, P73, P74, P75, P76, P77, P78, P79, P80, P81, P83, P84, P85, P86, P87, P88, P89, P90, P91, P93, P94, P95, P96, P97, P98, P99 |
| Microvascular disease | At least one is true:   - ICD-9: 35[45], 3572, 2504, 352, 36641, 36544, 36207, 2505, 7135, 2507, 58381, 2504 - ICD-10: E10[2345], E11[23456], E1[234][2345], G59, G60, G990, H35[01], H36, L984, M14[26], M908, N083 |
| Myocardial infarction | At least one is true:   - ICD-9: 410 - ICD-10: I21, I22, I252, I256 - ICPC-2: K75 |
| Nitrates | ATC: C01DA |
| Obesity | Body mass index ≥ 30 kg/m2 |
| Peripheral artery disease | At least one is true:   - Percutaneous Peripheral Revascularization - ICD-9: 44[014] - ICD-10: I702, I739, I74[234589] - ICPC-2: K92 |
| Possible Familial hypercholesterolemia | At least one is true:   - ICD-10: Z8342 - All are true - TC > 290 mg/dL or LDL-C > 190 mg/dL - MI ≤ 60 years in first-degree relative - MI ≤ 50 years in second-degree relative - TC > 290 mg/dL in adult first- or second-degree relative |
| RAAS Inhibitors | ATC: C09 |
| SCORE | SCORE, ESC 19 (0%; 1%; [2, 5[%; [5, 10[%; [10,[%) |
| Sex | Patient gender (male/female) |
| Smoking | More recent smoking status (current smoker/former smoker/never smoked) |
| Stable angina | At least one is true:   - ICD-9: 413, 4140 - ICD-10: I201, I208, I209, I251, I255 - ICPC-2: K76 |
| Stroke | At least one is true:   - ICPC-2: K89, K90 - Hemorrhagic or Ischemic Stroke |
| Structural heart disease | At least one is true:   - Left atrial volume index > 30 mL/m^2^ - Left atrial volume> 50 mL - Left atrial diameter > 38 mm - Interventricular septum thickness > 11 mm - Left ventricle posterior wall thickness > 11 mm - Left ventricular mass index > 115 g/m^2^ in males - Left ventricular mass index > 95 g/m^2^ in females |
| Transient ischemic attack | At least one is true:   - ICD-9: 435 - ICD-10: G45 - ICPC-2: K89 |
| Type 2 diabetes mellitus | At least one is true:   - HbA1c level ≥ 6.5% - Plasma glucose ≥ 200 mg/mL - Glucose lowering drugs except metformin in isolation |
| Unstable angina | At least one is true:   - ICD-9: 411 - ICD-10: I200 - ICPC-2: K74 |

UA - Unstable angina; MI - Myocardial infarction; SA - Stable angina; STK - Stroke; PAD - Peripheral arterial disease; LVEF - Left ventricular ejection fraction. **Diagnosis:** Primary care diagnoses are coded using ICPC-2 codes. Hospital diagnosis are coded using ICD-9 codes for all visits, except for hospitalization visits since 01-01-2017 that are coded using ICD-10; **Measurement:** Data comes from measurements performed at any venue care site; **Medication:** All prescribed medications from identified persons that match ATC codes. May include medications prescribed by providers outside ULSM.
